# A randomized experiment of the effects of food advertisements on food-related emotional expectancies in adults

**DOI:** 10.1177/13591053231168340

**Published:** 2023-04-15

**Authors:** Jenna R Cummings, Lindzey V Hoover, Ashley N Gearhardt

**Affiliations:** 1 University of Michigan, USA; 2 University of Liverpool, UK

**Keywords:** expectancy theory, food addiction, food advertisements, food-related emotional expectancies

## Abstract

Food-related emotional expectancies influence food intake, yet little is known about their determinants. The present study objectives were to experimentally test how food advertisements affect food-related emotional expectancies in adults and whether effects differed by individual levels of “food addiction” symptoms. Participants (*n* = 718; *M_age_* = 35.88, 36.8% with food addiction) were randomly assigned to watch video advertisements for highly processed foods, minimally processed foods, both food groups, or cellphones (control). Participants completed an attention check and questionnaires including the Anticipated Effects of Food Scale. Main effects of condition were non-significant. In participants with fewer symptoms of food addiction, watching video advertisements for highly processed foods increased expectancies that one would feel positive emotions while eating those foods, *B*(*SE*) = 0.40(0.16), *p* = 0.016, 95% CI (0.08, 0.72), *ΔR*^2^ = 0.03. Highly processed food advertisements may affect food-related emotional expectancies in adults who have not previously formed strong expectancies.

## Introduction

Many people overeat highly processed foods (e.g. cheeseburger sandwiches, fries, milkshakes), designed to be particularly rewarding through the addition of fat and/or refined carbohydrates (Schulte et al., 2015). Overeating highly processed foods displaces intake of minimally processed foods (e.g. berries, pecans, plain yogurt), which are more natural and contain health-promoting nutrients (Ayton et al., 2021). This pattern of food intake may result in lower diet quality, which robustly increases risk of non-communicable chronic diseases including cardiovascular disease, type 2 diabetes, and cancer (Schwingshackl et al., 2018). Applying psychological theory to the study of food intake may reveal critical information into why individuals overeat highly processed foods and undereat minimally processed foods. Understanding food intake is paramount given the high prevalence of non-communicable chronic diseases globally (Habib and Saha, 2010).

### Expectancy theory

A long-standing psychological theory applied to understand health-related behaviors is Expectancy Theory, which posits that through social and personal learning individuals develop memories of behavior outcomes, known as “expectancies,” and these expectancies impact their future behavior (Bandura, 1977; James, 1890; Tolman, 1949). For example, by watching others experience or by personally experiencing strong positive emotions when drinking alcohol, one may expect this outcome and be predisposed to drink more frequently and heavily (Goldman et al., 1999). Cross-sectional, longitudinal, and experimental evidence support the tenets of Expectancy Theory in explaining several health-related behaviors including alcohol, cigarette, and marijuana use (Buckner and Schmidt, 2008; Cohen et al., 2002; Montes et al., 2019; Morean et al., 2012; Scott-Sheldon et al., 2012) and disordered eating (Annus et al., 2008; Pearson et al., 2014).

Expectancy Theory may apply to other eating behaviors including overeating highly processed foods and undereating minimally processed foods. Food-related emotional expectancies—the anticipated positively- and negatively-valenced emotional outcomes of eating different foods (distinct from more general expectancies about eating)—may especially be relevant to understanding food intake (Cummings et al., 2020). In a cross-sectional study, stronger expectancies that one will feel positive emotions while eating highly processed foods and negative emotions while eating minimally processed foods were associated with greater intake of highly processed foods (Cummings et al., 2020). In a randomized experiment, increasing expectancies that one would feel positive emotions while eating highly processed food increased intake of those foods at the expense of minimally processed foods (Cummings et al., 2021).

### Determinants of food-related emotional expectancies

Although food-related emotional expectancies have explained patterns of food intake, little is known about their determinants. Research on alcohol expectancies suggests that expectancies begin forming in childhood and become relatively stable by adulthood (Dunn and Goldman, 1996; Smith and Goldman, 1994); however, the situational-specificity hypothesis proposes that relevant situations can shift expectancies after they are formed, influencing behavior in the moment (Wall et al., 2000). In support of this hypothesis, being in a bar versus neutral space led college students to have greater positive alcohol expectancies (Monk and Heim, 2013; Wall et al., 2000, 2001). Being in a simulated fast-food restaurant caused college students to have greater positive highly-processed food expectancies (Cummings et al., 2021), yet no other situational influences on food-related emotional expectancies have been explored.

Food advertising, which is an activity an organization engages in to facilitate an exchange between its food products and customers, is widespread (Story and French, 2004) and may impact food-related emotional expectancies. Food-related organizations in the United States are the leading buyers of television, newspaper, magazine, billboard, and radio advertising (Story and French, 2004), spending almost $13 billion per year (UConn Rudd Center for Food Policy and Obesity, 2017). More than 80% of food advertising promotes highly processed foods (UConn Rudd Center for Food Policy and Obesity, 2017), although there is public interest in minimally processed food advertising as a strategy to improve diet quality (Kraak et al., 2006). Regardless of food type, marketers design food advertisements to promote viewers’ association of positive emotions with the specific food advertised (Page and Brewster, 2009). As a result, highly and minimally processed food advertisements may shift corresponding food-related emotional expectancies to be more positive and less negative.

### Individual differences

Food advertisements may differentially affect food-related emotional expectancies in individuals with and without “food addiction,” a phenotype marked by strong cravings for highly processed foods, diminished control over their intake, and overconsumption despite negative consequences including clinically-significant distress and diet-related disease (Schulte and Gearhardt, 2017; Schulte et al., 2015). Individuals with elevated food addiction symptoms hold stronger food-related emotional expectancies, particularly expectancies that one will feel positive emotions while eating highly processed foods and negative emotions while eating minimally processed foods (Cummings et al., 2020). In response to highly processed food images and anticipated receipt of highly processed food, individuals with food addiction have shown greater neural activation in reward-related regions compared to control (Gearhardt et al., 2011; Schulte et al., 2019). This suggests highly processed food advertisements may especially amplify positive and reduce negative expectancies regarding eating highly processed foods in individuals with a greater number of food addiction symptoms. Alternatively, food advertisements may not impact food-related emotional expectancies in these individuals because their existing expectancies are strong and potentially less malleable.

### The present research

The primary aim of the present study was to test effects of food advertisements on food-related emotional expectancies. We hypothesized that (a) highly processed food advertisements would increase positive and decrease negative highly-processed food expectancies and would not affect minimally processed food expectancies; (b) minimally processed food advertisements would increase positive and decrease negative minimally-processed food expectancies and would not affect highly processed food expectancies; and (c) a mixture of highly and minimally processed food advertisements would increase positive and decrease negative highly- and minimally-processed food expectancies. The secondary aim was to test the moderating role of food addiction symptoms. We hypothesized that highly processed food advertisements would increase positive and decrease negative highly-processed food expectancies to a greater extent in individuals with a greater number of food addiction symptoms.^1^

## Materials and methods

### Transparency and openness

We preregistered the aims, hypotheses, and method on the Open Science Framework prior to conducting the research, reducing experimenter bias: https://osf.io/mep23. We report how we determined our sample size and all data exclusions, manipulations, and measures in the study.

### Design

The study design was a four-level [video advertisements for highly processed foods, minimally processed foods, both food groups, or cellphones (control)] randomized between-subjects experiment. Video advertisements for cellphones were selected as the exposure for the control condition because cellphones (like food) are frequently advertised and widely purchased (Harris, 2013); however, cellphones are non-ingestible products.

### Participants

Participants were recruited August 11th–12th, 2020 via Amazon’s Mechanical Turk for “A Study on Advertisements and Beliefs” in which they would, “watch and rate the effectiveness of four short advertisement clips and complete some questionnaires about beliefs, feelings, and typical behaviors.” A power analysis conducted in G*Power Version 3.1.9.6 with the following parameters determined a sample size of 788 participants: one-way analysis of variance (ANOVA) with four groups, power = 0.90, *α* = 0.05, and a small expected effect size (Cohen’s *d* = 0.29). The expected effect size was based on prior work demonstrating exposure to a simulated fast-food restaurant increased positive highly-processed food expectancies (Cummings et al., 2021). The power analysis yielded a sample size of 608 but, due to available research funds, 180 participants were added to mitigate anticipated non-compliance.

A total of 947 participants started the study before expiration. Inclusion criteria were ⩾18 years old, living in the United States, and ⩾99% approval rating by other investigators. Data of a participant were excluded from analysis if a participant did not complete the study (*n* = 36) or at least one of the following preregistered exclusion criteria were met: participant completed the study in <3 minutes (*n* = 2), participant incorrectly answered the attention check question about what kind of advertisements they watched (*n* = 56), participant reported improbable values for adult height and weight (i.e. body mass index <12 or >70; *n* = 125), and participant incorrectly answered quality control questions (e.g. “What year is it?”) at the end of the study (*n* = 10). The analytic sample therefore included 718 participants. Demographics of the analytic sample are provided in Table 1.

**Table 1.** Demographics of the analytic sample.

	Total (*n* = 718)	Phone Advertisements (*n* = 174)	HP Food Advertisements (*n* = 185)	MP Food Advertisements (*n* = 183)	HP + MP Food Advertisements (*n* = 176)	*F* or *Χ*^2^	*p*	*η*^2^ or *Φ*
Age (*M*, *SD*)	35.88 (11.40)	37.02(11.42)	36.60(12.66)	34.33(10.08)	35.61(11.18)	1.98	0.116	0.01
Gender (*n*, *%*)						8.59	0.476	0.11
Woman	295 (41.1%)	77 (44.3%)	69 (37.3%)	75 (41.0%)	74 (42.0%)			
Man	416 (57.9%)	97 (55.7%)	112 (60.5%)	106 (57.9%)	101 (57.4%)			
Trans	0 (0.0%)	0 (0.0%)	0 (0.0%)	0 (0.0%)	0 (0.0%)			
Fluid	2 (0.3%)	0 (0.0%)	1 (0.5%)	0 (0.0%)	1 (0.6%)			
Prefer not to answer	5 (0.7%)	0 (0.0%)	3 (1.6%)	2 (1.1%)	0 (0.0%)			
Race/ethnicity (*n*, *%*)						13.20	0.587	0.14
Native American or Alaskan	50 (7.0%)	9 (5.2%)	20 (10.8%)	11 (6.0%)	10 (5.7%)			
Hawaiian or Pacific Islander	0 (0.0%)	0 (0.0%)	0 (0.0%)	0 (0.0%)	0 (0.0%)			
Asian American	40 (5.6%)	9 (5.2%)	10 (5.4%)	14 (7.7%)	7 (4.0%)			
Black or African American	164 (22.8%)	39 (22.4%)	40 (21.6%)	45 (24.6%)	40 (22.7%)			
White or European American	401 (55.8%)	103 (59.2%)	96 (51.9%)	97 (53.0%)	105 (59.7%)			
Hispanic/Latinx	40 (5.6%)	11 (6.3%)	13 (7.0%)	9 (4.9%)	7 (4.0%)			
Prefer not to answer	23 (3.2%)	3 (1.7%)	6 (3.2%)	7 (3.8%)	7 (4.0%)			
Education (*n*, *%*)						20.10	0.327	0.17
Less than high school	1 (0.1%)	0 (0.0%)	0 (0.0%)	0 (0.0%)	1 (0.6%)			
High school graduate	37 (5.2%)	10 (5.7%)	8 (4.3%)	10 (5.5%)	9 (5.1%)			
Some college	85 (11.8%)	21 (12.1%)	23 (12.4%)	21 (11.5%)	20 (11.4%)			
Associates degree	31 (4.3%)	14 (8.0%)	2 (1.1%)	7 (3.8%)	8 (4.5%)			
Bachelors degree	419 (58.4%)	101 (58.0%)	115 (62.2%)	104 (56.8%)	99 (56.3%)			
Advanced degree	139 (19.4%)	27 (15.5%)	34 (18.4%)	39 (21.3%)	39 (22.2%)			
Prefer not to answer	6 (0.8%)	1 (0.6%)	3 (1.6%)	2 (1.1%)	0 (0.0%)			
BMI (*M*, *SD*)	24.72 (6.91)	25.57 (7.44)	24.55 (6.79)	24.06 (6.38)	24.77 (6.99)	1.23	0.300	0.01
mYFAS 2.0						0.90	0.825	0.04
No food addiction	402 (63.2%)	99 (63.1%)	100 (65.4%)	101 (60.5%)	102 (64.2%)			
Food addiction	234 (36.8%)	58 (36.9%)	53 (34.6%)	66 (39.5%)	57 (35.8%)			
Number of food addiction symptoms (*M*, *SD*)	4.70 (4.17)	4.43 (4.08)	4.65 (4.16)	4.84 (4.25)	4.85 (4.19)	0.36	0.779	0.00

HP: highly processed; MP: minimally processed; BMI: Body Mass Index; mYFAS 2.0 = Modified Yale Food Addiction Scale 2.0.

### Procedure

The University of Michigan Institutional Review Board termed the study exempt (HUM00186009). Participants answered a question to ensure they were ⩾18 years old and provided informed consent. Participants were next randomly assigned via a randomizer in survey flow to watch video advertisements for highly processed foods, minimally processed foods, both food groups, or cellphones (control).

In each condition, participants viewed four 15-second video advertisements back-to-back. Participants were unable to move to the next survey page until 60 seconds passed after clicking play. In the highly processed foods condition, participants viewed commercials for McDonald’s Quarter Pounders©; McDonald’s dipping sauces and McNuggets©; Wendy’s Bacon Deluxe, Baconator, and Bacon and Blue©; and Wendy’s Jr. Frosty©. In the minimally processed foods condition, participants viewed commercials for McDonald’s Fruit and Yogurt Parfait©; McDonald’s Southwest Salad©; Wendy’s Apple Pecan Chicken Salad©; and Wendy’s Harvest Apple Chicken Salad©. In the both food groups condition, participants viewed commercials for McDonald’s Fruit and Yogurt Parfait©; McDonald’s Quarter Pounders©; Wendy’s Harvest Apple Chicken Salad©; and Wendy’s Jr. Frosty©. In the control condition, participants viewed commercials for cellphones with AT&T Mobile Share©; HTC Aria©; HTC Imagio©; and AT&T Samsung Galaxy©. All commercials focused on the foods/cellphones and did not feature fully visible people interacting with the foods/cellphones (a few showed hands interacting with the foods/cellphones).^2^

After watching the video advertisements, participants completed an attention check in which they were asked what kind of advertisements they just watched and then completed the Anticipated Effects of Food Scale. Next, they completed the modified Yale Food Addiction Scale 2.0 and additional measures in randomized order and answered demographic questions. Participants had the option to select *Prefer* *not to answer* for questions. Before receiving compensation, participants answered quality control questions. Participants received $1.00 for study completion, completing in an average of 14.19 (*SD* = 7.48) minutes.

### Measures

#### Anticipated Effects of Food Scale

The 62-item Anticipated Effects of Food Scale measures food-related emotional expectancies (Cummings et al., 2020). Participants were asked to imagine eating highly processed foods (e.g. sweets, salty snacks, fast foods, sugary drinks) and rate how much they expect to feel 31 positive (e.g. relaxed, cheerful, glad) and negative (e.g. disgusting, worried, frustrated) emotions while eating. Participants were then asked to imagine eating minimally processed foods (e.g. fruit, vegetables) and rate how much they expect to feel the same 31 positive and negative emotions while eating. Participants rated items on a 6-point Likert scale from 1 (*Definitely Not*) to 6 (*Definitely*). The scale yielded four food expectancy subscales by separately averaging positive and negative emotion ratings for each food group [positive highly-processed food expectancies (α = 0.95), negative highly-processed food expectancies (α = 0.96), positive minimally-processed food expectancies (α = 0.94), and negative minimally-processed food expectancies (α = 0.97)].

#### Modified Yale Food Addiction Scale 2.0

The 13-item modified Yale Food Addiction Scale 2.0 measures addictive-like responses to highly processed food based on the Diagnostic and Statistical Manual of Mental Disorders (5th ed.) criteria for substance use disorders (Schulte and Gearhardt, 2017). The instructions are, “People sometimes have difficulty controlling how much they eat of certain foods such as: **sweets** like ice cream, chocolate, doughnuts, cookies, cake, candy, **starches** like white bread, rolls, pasta, and rice, **salty snacks** like chips, pretzels, and crackers, **fatty foods** like steak, bacon, hamburgers, cheeseburgers, pizza, and French fries, and **sugary drinks** like soda pop, lemonade, sports drinks, and energy drinks. When the following questions ask about “CERTAIN FOODS” please think of ANY foods or beverages similar to those listed in the food or beverage groups above or ANY OTHER foods you have had difficulty with in the past year” Sample items include, “Eating the same amount of food did not give me as much enjoyment as it used to,” and “I ate to the point where I felt physically ill.” Participants rated each item on an 8-point Likert scale from 1 (*Never*) to 8 (*Every Day*), and whether this rating met the “diagnostic” threshold for each symptom was determined. All symptom values were summed to create a dimensional score of food addiction (*α* = 0.93). For descriptive purposes, individuals were also dichotomously categorized based on whether they met criteria for a food addiction “diagnosis” (i.e. endorsed two or more symptoms plus impairment or distress).

#### Additional measures

Participants completed the Restrained Eating subscale of the Dutch Eating Behavior Questionnaire, which includes 10 items (e.g. “Do you try to eat less at mealtimes than you would like to eat?”) (van Strien et al., 1986). Participants answered questions about their familiarity with, liking of, likelihood of buying products from, and identifying with McDonald’s, Wendy’s, AT&T, Samsung, Verizon, and HTC. Participants reported how often they eat food from McDonald’s, Wendy’s, and any place where you can buy fast food (food eaten there or carried out).

#### Demographic questions

Participants reported their age, gender, race/ethnicity, highest level of completed education, height, and weight. Body mass index was calculated using the standard formula (kg/m^2^).

### Statistical analysis

For the primary aim, four Analyses of Variance (ANOVA) were conducted with positive and negative highly- and minimally-processed food expectancies as dependent variables. Experimental condition was the independent variable/between-subjects factor. Post hoc Fisher’s Least Significant Difference (LSD) tests were conducted for pairwise comparisons.

For the secondary aim, hierarchical multiple regressions were conducted with positive and negative highly- and minimally-processed food expectancies as dependent variables. Independent variables included experimental condition (dummy coded into three variables with the control condition as the reference group) in Step 1, food addiction symptoms in Step 2, and their interaction (coded by multiplicative interaction terms) in Step 3. Significant interactions were followed up with tests of simple effects; subgroups were created with a median split on food addiction symptoms [<4 symptoms = 0 (47.0% of sample), ⩾ 4 symptoms = 1 (53.0% of sample)].

Analysis was conducted in SPSS, Build 1.0.0.1447 (IBM Corporation, Armonk, NY) using default procedures to account for missing data (<3% of data were missing). Significance was set at *p* < 0.05.

## Results

Table 1 presents univariate statistics for variables of interest. Differences in participant age, gender, race/ethnicity, education, body mass index, and food addiction symptoms and diagnosis across experimental conditions were non-significant. On average, participants met the threshold for 4.70 out of 11 symptoms of food addiction, with 36.8% of the sample meeting criteria for food addiction.

Table 2 presents estimates from ANOVA. There were no significant differences in positive and negative highly- and minimally-processed food expectancies across experimental conditions. Post hoc LSD indicated no significant pairwise comparison between conditions (*p*s >0.08).

**Table 2.** Means and standard deviations of food expectancies overall and by experimental condition and ANOVA estimates.

	Total (*n* = 718)	Phone Advertisements (*n* = 174)	HP Food Advertisements (*n* = 185)	MP Food Advertisements (*n* = 183)	HP+MP Food Advertisements (*n* = 176)	*F*	*p*	*η* ^2^	Cohen’s *d*
	*M* (*SD*)	*M* (*SD*)	*M* (*SD*)	*M* (*SD*)	*M* (*SD*)				
+HP Food Expectancies	4.07 (1.14)	4.00 (1.18)	4.20 (1.10)	4.07 (1.10)	3.99 (1.18)	1.30	0.272	0.01	0.14
–HP Food Expectancies	3.42 (1.34)	3.41 (1.34)	3.46 (1.37)	3.41 (1.29)	3.38 (1.37)	0.11	0.954	0.00	0.04
+MP Food Expectancies	4.33 (1.02)	4.32 (0.99)	4.29 (1.07)	4.39 (1.01)	4.31 (1.01)	0.35	0.791	0.00	0.06
–MP Food Expectancies	3.01 (1.51)	2.92 (1.57)	3.12 (1.49)	2.96 (1.47)	3.05 (1.53)	0.63	0.598	0.00	0.11

HP: highly processed; MP = minimally processed; +: positive; *–*: negative.

**Figure 1.**
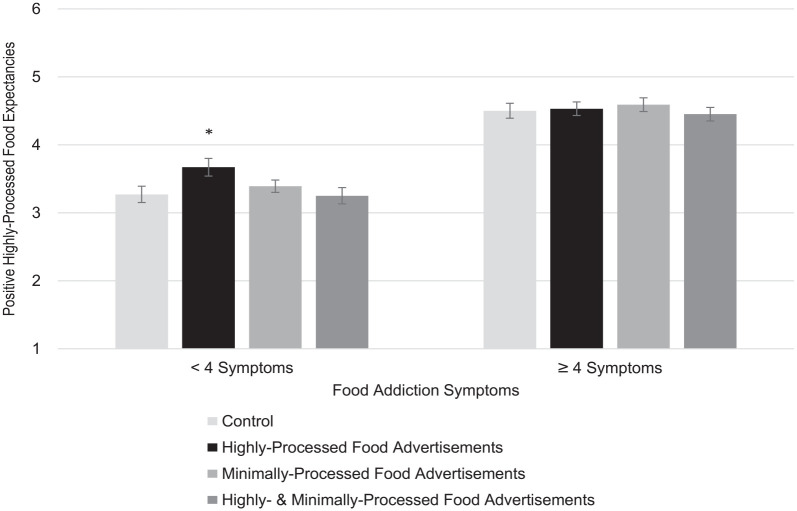
Simple effects of food advertisements on positive highly-processed food expectancies by food addiction symptoms. *p < .05.

Table 3 presents estimates from hierarchical multiple regressions. For positive highly-processed food expectancies, the association with experimental condition was non-significant in Step 1. In Step 2, a greater number of food addiction symptoms was associated with stronger positive highly-processed food expectancies, accounting for 32% of the variance. In Step 3, there was a significant interaction between food addiction symptoms and the highly processed food advertisements condition. Simple effects analysis revealed that, in participants with fewer food addiction symptoms, watching video advertisements for highly processed foods increased positive highly-processed food expectancies compared to control, *B*(*SE*) = 0.40(0.16), *p* = 0.016, 95% CI (0.08, 0.72), *ΔR*^2^ = 0.03 (illustrated in Figure 1). In participants with a greater number of food addiction symptoms, there were no significant differences in positive highly-processed food expectancies after watching video advertisements for highly processed foods compared to control, *B*(*SE*) = 0.04(0.15), *p* = 0.800, 95% CI (−0.25, 0.33), *ΔR*^2^ = 0.00.

**Table 3.** Hierarchical multiple regressions estimates.

	*B (SE)*	*p*	95% CI (Lower, Upper)	*ΔR* ^2^
	+HP Food Expectancies			
Step 1: Experimental Condition				0.01
HP Food Advertisements	0.21(0.13)	0.096	(−0.04, 0.46)	
MP Food Advertisements	0.11(0.12)	0.374	(−0.13, 0.36)	
HP+MP Food Advertisements	−0.02(0.13)	0.871	(−0.27, 0.23)	
Step 2: Food Addiction Symptoms				0.32
mYFAS 2.0	0.15(0.01)	<0.001	( 0.14, 0.17)	
Step 3: Interaction				0.01
HP Food Advertisements*mYFAS 2.0	−0.05(0.03)	0.034	(−0.10, 0.00)	
MP Food Advertisements*mYFAS 2.0	−0.02(0.03)	0.375	(−0.07, 0.03)	
HP+MP Food Advertisements*mYFAS 2.0	−0.03(0.03)	0.251	(−0.08, 0.02)	
	−HP Food Expectancies			
Step 1: Experimental Condition				0.00
HP Food Advertisements	0.01(0.15)	0.953	(−0.28, 0.30)	
MP Food Advertisements	0.08(0.14)	0.574	(−0.20, 0.36)	
HP+MP Food Advertisements	−0.04(0.14)	0.794	(−0.32, 0.25)	
Step 2: Food Addiction Symptoms				0.36
mYFAS 2.0	0.19(0.01)	<0.001	(0.17, 0.20)	
Step 3: Interaction				0.00
HP Food Advertisements*mYFAS 2.0	−0.05(0.03)	0.083	(−0.11, 0.01)	
MP Food Advertisements*mYFAS 2.0	−0.02(0.03)	0.594	(−0.07, 0.04)	
HP+MP Food Advertisements*mYFAS 2.0	−0.04(0.03)	0.158	(−0.10, 0.02)	
	+MP Food Expectancies			
Step 1: Experimental Condition				0.00
HP Food Advertisements	−0.02(0.11)	0.879	(−0.24, 0.21)	
MP Food Advertisements	0.08(0.11)	0.458	(−0.14, 0.30)	
HP+MP Food Advertisements	−0.03(0.11)	0.825	(−0.25, 0.20)	
Step 2: Food Addiction Symptoms				0.13
mYFAS 2.0	0.09(0.01)	<0.001	( 0.07, 0.10)	
Step 3: Interaction				0.01
HP Food Advertisements*mYFAS 2.0	−0.03(0.03)	0.215	(−0.08, 0.02)	
MP Food Advertisements*mYFAS 2.0	0.01(0.03)	0.656	(−0.04, 0.06)	
HP + MP Food Advertisements*mYFAS 2.0	−0.03(0.03)	0.176	(−0.08, 0.02)	
	−MP Food Expectancies			
Step 1: Experimental Condition				0.00
HP Food Advertisements	0.19(0.16)	0.235	(−0.13, 0.52)	
MP Food Advertisements	0.15(0.16)	0.351	(−0.17, 0.46)	
HP+MP Food Advertisements	0.13(0.16)	0.415	(−0.19, 0.45)	
Step 2: Food Addiction Symptoms				0.53
mYFAS 2.0	0.25(0.01)	< 0.001	( 0.23, 0.27)	
Step 3: Interaction				0.00
HP Food Advertisements*mYFAS 2.0	−0.05(0.03)	0.077	(−0.10, 0.01)	
MP Food Advertisements*mYFAS 2.0	−0.02(0.03)	0.448	(−0.07, 0.03)	
HP+MP Food Advertisements*mYFAS 2.0	−0.02(0.03)	0.361	(−0.08, 0.03)	

HP: highly processed; MP: minimally processed; mYFAS 2.0: Modified Yale Food Addiction Scale 2.0; +: positive; *–*: negative.

For negative highly-processed and positive and negative minimally-processed food expectancies, associations with experimental condition were non-significant in Step 1. In Step 2, a greater number of food addiction symptoms was associated with stronger negative highly-processed, positive minimally-processed, and negative minimally-processed food expectancies, accounting for 36%, 13%, and 53% of the variances, respectively. In Step 3, interactions between food addiction symptoms and experimental condition were non-significant.

## Discussion

Little is known about the determinants of food-related emotional expectancies, which is a critical gap because of the influence of these expectancies on food intake. This was the first study (to our knowledge) to test effects of food advertisements on food-related emotional expectancies. There was much strength in the study design including transparency and openness, a large sample of adults, randomization to groups, inclusion of a control group, and use of ecologically valid advertisements for highly and minimally processed foods.

In individuals with fewer symptoms of food addiction, viewing highly processed food video advertisements increased expectancies that one would feel positive emotions while eating those foods. Prior work shows individuals with fewer food addiction symptoms hold weaker food-related emotional expectancies (Cummings et al., 2020), whereas individuals with greater symptoms hold much stronger expectancies, a finding replicated in the present study. Highly processed food advertisements may therefore only situationally affect food-related emotional expectancies in adults who have not previously formed strong expectancies. Although a small effect size was observed in the present study, advertisements for highly processed foods are wide-reaching in the United States (Story and French, 2004; UConn Rudd Center for Food Policy and Obesity, 2017), which suggests there could be a larger net effect of highly processed food advertisements on food-related emotional expectancies in adults with few symptoms of food addiction.

Main effects of food advertisements on food-related emotional expectancies, however, were non-significant. Although being in a bar and a simulated fast-food restaurant led college students to have greater positive alcohol (Monk and Heim, 2013; Wall et al., 2000, 2001) and highly-processed food (Cummings et al., 2021) expectancies, respectively, there may be boundary conditions to situations affecting expectancies across individuals. Entering a built environment with an array of stimuli and food cues is more immersive than viewing video advertisements, and the former may more consistently influence food-related emotional expectancies for adults regardless of their previously formed expectancies. It is also possible that repeated exposure to food advertisements over time exerts a stronger influence on food-related emotional expectancies compared to a one-time situational effect. Future longitudinal research examining cumulative exposures to food advertisements and subsequent changes in food-related emotional expectancies could test this possibility.

Food advertisements may also have a greater impact on food-related emotional expectancies during adolescence or childhood, when expectancies may be especially malleable (Dunn and Goldman, 1996; Smith and Goldman, 1994). Prior experiments have shown that viewing film clips depicting desirable outcomes of alcohol use increased positive alcohol expectancies in adolescents (Kulick and Rosenberg, 2001), and viewing video advertisements for beer increased positive alcohol expectancies in fourth to fifth grade children (Dunn and Yniguez, 1999; *c.f.* Lipsitz et al., 1993). Future research should incorporate a developmental perspective in research on food advertisements and food-related emotional expectancies. Food-related emotional expectancies may mediate the consistently-observed stimulatory effect of food advertisements on food intake in children (Boyland et al., 2016).

Identifying potential determinants of food-related emotional expectancies including food advertisements is important for policy makers and practitioners. Successful interventions on other health-related behaviors have indirectly targeted those behaviors through influencing emotional expectancies. For example, a meta-analysis of 23 studies indicates the efficacy of reducing alcohol use in high school and college students through reducing positive alcohol expectancies (Gesualdo and Pinquart, 2021). Knowledge on how to influence food-related emotional expectancies will shed light on dietary intervention strategies (e.g. policies limiting food advertisements, therapy including expectancy challenge).

Readers should consider study limitations when interpreting results. Video advertisements for highly and minimally processed foods were from fast food companies; it is possible that video advertisements for minimally processed foods from companies that only sell minimally processed foods may have different effects on food-related emotional expectancies. Companies like these occupy less than 5% of the food advertising landscape (UConn Rudd Center for Food Policy and Obesity, 2019), yet future research should test the effects of video advertisements from these companies and consider study designs that disentangle the effects of branding versus food cues on food-related emotional expectancies. The available measurement method for food-related emotional expectancies is a self-report questionnaire, yet it is possible the effect of food advertisements on food-related emotional expectancies would be differentially captured by an implicit measure of expectancies like an implicit association test (Reich et al., 2010). It is also possible the food expectancy measure’s 6-point Likert scale restricted responses for some individuals (albeit responses were normally distributed with means around the midpoint of the scale). Particularly for clinical samples with higher rates of food addiction, widening response options may increase sensitivity to detecting situational fluctuations in food-related emotional expectancies. The present study did not include measurement of food intake, precluding tests of the effects of food advertisements on food intake mediated by food-related emotional expectancies. Future studies on determinants of food-related emotional expectancies could measure food intake or food choice (if online; Schreiber et al., 2020) to test for mediation. While Amazon’s Mechanical Turk platform allowed for recruitment of more demographically-varied participants than traditional approaches (e.g. University subject pool) (Berinsky et al., 2012), data quality may be limited. Following principles of web survey design (e.g. designing surveys to be no longer than necessary, including several data quality checks)—as was done in the present study—improves data quality (Hauser et al., 2019). Nonetheless, the generalizability of the results may be limited due to the crowdsourcing approach and researchers should consider data collection via alternatives to Amazon’s Mechanical Turk in light of concerns with the platform (Webb and Tangney, 2022). Although the sample size was large, with over a third of the sample meeting criteria for food addiction, the sample may have been underpowered to detect all moderation effects.

Limitations notwithstanding, findings from this experiment are novel and contribute to knowledge on the determinants of food-related emotional expectancies. For adults who have not previously formed strong food-related emotional expectancies, video advertisements for highly processed foods may have a stimulatory effect on expectations that one would feel positive emotions while eating those foods. These findings are important for public health because of the pervasiveness of highly processed food advertisements and the role of expectancies in shaping behavior.

## Notes

1. The research was preregistered in an independent registry with an analysis plan: https://osf.io/mep23. Our secondary aims also included tests of the moderating role of race/ethnicity, socioeconomic status (indicated by highest education level), and dietary restraint (measured by averaging the items from the Restrained Eating subscale from the Dutch Eating Behavior Questionnaire; *α* = 0.93) in the effects of food advertisements on food-related emotional expectancies. Our hypotheses that advertisements for highly processed food would increase positive and decrease negative highly-processed food expectancies to a greater extent in Black Americans, individuals with less education, and individual scoring higher in dietary restraint were not supported. In post hoc analysis suggested by a reviewer, we tested the moderating role of gender in the effects of food advertisements on food-related emotional expectancies. Gender did not significantly moderate the effects of food advertisements on positive and negative highly-processed, and positive minimally-processed, food expectancies. Gender significantly moderated the effect of food advertisements on negative minimally-processed food expectancies, *F* = 4.81, *p* = 0.003, *η*^2^ = 0.02, Cohen’s *d* = 0.29. In women, compared to the control condition [*M(SE)* = 2.39(0.17)], watching video advertisements for highly processed foods [*M(SE)* = 3.10(0.18), *p* = 0.004] and watching video advertisements for minimally processed foods [*M(SE)* = 2.99(0.17), *p* = 0.013] increased expectancies that one would feel negative emotions while eating minimally processed foods. The data and syntax for these tests are available online via the Open Science Framework: https://osf.io/qxy7w/.

2. In accordance with copyright laws, we share the links to commercials available on YouTube©: McDonald’s dipping sauces and McNuggets©: https://youtu.be/vO1VjeGNyT4; Wendy’s Jr. Frosty©: https://youtu.be/Bo4Xsys-sto; and HTC Imagio©: https://youtu.be/vu6qkTVAjLg. Please contact the corresponding author regarding commercials unavailable on YouTube©.
